# Fabrication of the heterojunction catalyst BiVO_4_/P25 and its visible-light photocatalytic activities

**DOI:** 10.1098/rsos.180752

**Published:** 2018-08-15

**Authors:** Heshan Cai, Linmei Cheng, Feng Xu, Hailong Wang, Weicheng Xu, Fuhua Li

**Affiliations:** Department of Transportation and Civil Architecture, Foshan University, Foshan 528000, People's Republic of China

**Keywords:** BiVO_4_/P25, visible light photocatalyst, one-step hydrothermal

## Abstract

A heterojunction catalyst, BiVO_4_/P25, was successfully fabricated using a one-step hydrothermal method. The prepared composite was characterized using XRD, XPS, Raman, FT-IR, UV–vis, SEM, HRTEM and PL. The HRTEM pictures revealed that the heterostructured composite was composed of BiVO_4_ and P25, and from the pictures of SEM we could see the P25 nanoparticles assembling on the surface of flower-shaped BiVO_4_ nanostructures. The XPS spectra showed that the prepared catalyst consisted of Bi, V, O, Ti and C. The photocatalytic activity of BiVO_4_/P25 was evaluated by degraded methyl blue (MB) and tetracycline under visible light illumination (*λ* > 420 nm), and the results showed that BiVO_4_/P25 composite has a better photocatalytic performance compared with pure BiVO_4_ and the most active c-BiVO_4_/P25 sample showed enough catalytic stability after three successive reuses for MB photodegradation. The enhanced photocatalytic performance could mainly be attributed to the better optical absorption ability and good absorption ability of organic contaminants.

## 1. Introduction

Photocatalysis has attracted intensive attention as a cost-effective, green chemical technology [1]. Visible light responding materials, such as BiVO_4_, ZnWO_4_ and AgVO_4_ were the promising semiconductors which had been previously applied to photocatalysis and have gained a great deal of attention [2]. Bismuth-based semiconductors (e.g. Bi_2_WO_6_, BiVO_4_, Bi_4_Ti_3_O_12_, Bi_2_O_2_CO_3_, BiOI, BiOBr and Bi_2_MoO_6_) as new types of photocatalytic materials have attracted great attention [3]. Bismuth vanadate (BiVO_4_) has three phases, which include tetragonal zircon, tetragonal scheelite and monoclinic scheelite structures [4–6]. Among these three phases, the monoclinic scheelite bismuth vanadate (m-BiVO_4_) has displayed the best photocatalytic ability. This has been found to be due to its narrow band gap (*E*_g_) of about 2.4 eV, visible light photocatalytic ability, good stability, and environmental friendliness. Furthermore, m-BiVO_4_ has also been used in water splitting, organic coatings, and so on [7–9]. It is believed that m-BiVO_4_ could potentially address the dangers of the energy crisis and environmental problems of modern society [10]. Therefore, increasing amount of attention has been given to it. However, single BiVO_4_ has some deficiencies, such as low photo-quantum efficiency, low interfacial charge-transfer rates and high recombination of photo-induced electron pairs [11–13]. All of the aforementioned drawbacks have tended to limit its development and application [14–18]. Many previous studies have focused on the improvements of the visible light-driven abilities of BiVO_4_, such as different morphologies, heterojunction, element doping, and so on. Many of the previous studies have reported that the heterojunction structures could make up for the deficiency of the single m-BiVO_4_. Therefore, this has become a hot field of study in photocatalysis due to the potential advantages [19–22]. The heterojunction uses the different structures of a conduction band (CB) and valence band (VB) in order to induce the transfer of a photon-generated carrier, as well as to enhance its separation efficiency. This has been found to result in high photocatalytic performances [23–25]. In recent years, there have been many documented successful cases of BiVO_4_-based heterojunctions. These included: Ag_2_CO_3_/BiVO_4_; Ag/Ag_2_O–BiVO_4_; BiVO_4_:Ag; BiVO_4_/BiOCl; BiVO_4_/BiOBr; BiPO_4_/BiVO_4_; Fe_2_O_3_/3DOM BiVO_4_; BiVO_4_/InVO_4_; BiVO_4_/Bi_2_WO_6_; BiVO_4_/Bi_2_Ti_2_O_7_; m-BiVO_4_/t-BiVO_4_, and so on [17–28]. Also, carbonaceous materials, such as activated carbon (AG), carbon dots, fullerene (C_60_), carbon nanotubes and graphene have been widely employed for enhancement of photocatalytic activity of BiVO_4_ [29]. Graphitic carbon nitride (g-C_3_N_4_) as a metal-free polymeric semiconductor doped on a Bi-based composite could improve the photocatalytic performance of Bi-based materials [30,31]. Xue *et al*. [32] fabricated a ternary heterostructured g-C_3_N_4_/Ag/BiVO_4_ by a facile *in situ* precipitation method and exhibited higher photocatalytic activity than pure g-C_3_N_4_, BiVO_4_ and Ag/BiVO_4_. Gu *et al*. synthesized a CeO*_χ_*/BiVO_4_ heterostructure using hydrothermal and ion-impregnation method, the results showed that 5.7 wt% of CeO*_χ_*/BiVO_4_ exhibited the highest photocatalytic activity [33]. Lopes *et al*. [34] successfully synthesized an m-BiVO_4_/t-BiVO_4_ heterostructure using a hydrothermal method, which used the different crystal phases of the BiVO_4_ to form a heterojunction. The results showed that the photocatalytic ability had been improved when compared to that of the pure m-BiVO_4_ and pure t-BiVO_4_. All of these findings demonstrated that the photocatalytic ability of a heterojunction was superior to that of a single catalyst due to the high separation rate of the photon-generated carrier.

This study synthesized a BiVO_4_/P25 heterostructure using a one-step hydrothermal method. Then, the different dosage amounts of P25 were compared in order to draw conclusions. It was determined that when the amount of P25 was 0.15 g, the heterojunction photocatalytic abilities of the BiVO_4_/P25 were evident. When the amount of P25 was increased, the adsorbing performance was observed to have improved. However, the photocatalytic abilities and recycling utilization had receded. The results obtained confirmed that the P25 displayed an important role in the BiVO_4_/P25 heterostructure. Also, the degradation rate of the methyl blue (MB) had reached up to 85% in 60 min under a 550 W xenon lamp illumination (*λ* > 420 nm), and the photocatalytic efficiency had been improved by more than 75% when compared with the pure BiVO_4_.

## 2. Material and methods

### 2.1. Materials

All chemicals were obtained from commercial sources and used without further purification. Starting materials include bismuth nitrate pentahydrate (Bi(NO_3_)_3_ · 5H_2_O, Kemiou, 99%), ammonium metavanadate (NH_4_VO_3_, Fuchen, 99%), sodium hydroxide (NaOH, Xilong, 96.0%), ethanol (Fuyu, 100%), nitric acid (HNO_3_, Guanghua, 67%), nano-titanium dioxide (P25, Macklin, AR) and the deionized water was made during the experimental time period.

### 2.2. Preparation of BiVO_4_

The BiVO_4_ nanocrystals were synthesized via a hydrothermal method. A solution of Bi(NO_3_)_3_·5H_2_O (2.45 g) in HNO_3_ solution (4 mol l^−1^, 40 ml) was added to a solution of NH_4_VO_3_ (0.6 g) in NaOH solution (4 mol l^−1^, 40 ml). The mixture was stirred for 20 min and sonicated for 10 min. The pH was adjusted to 9. Then, the solution was left overnight. The mixture solution was transferred to a Teflon-lined stainless-steel autoclave and maintained at 180°C for 12 h. The resultant products were filtered, washed with deionized water and ethanol. Finally, the yellow product was dried at 80°C for 6 h in a vacuum oven.

### 2.3. Synthesis of the BiVO_4_/P25 heterojunction catalysts

The BiVO_4_/P25 nanocomposite was synthesized using a one-step hydrothermal method. The steps of the method were as follows: 2.45 g of Bi(NO_3_)_3_·5H_2_O and 0.6 g of NH_4_VO_3_ were added to 40 ml of a 4 mol l^−1^ HNO_3_ solution and NaOH solution, respectively, which were referred to in this study as Solution A and Solution B. Then, 0.05, 0.1, 0.15 and 0.2 g of P25 were added to Solution A, and the two solutions were mixed. Magnetic stirring was conducted for 20 min, and sonication was performed for the dissolving process for another 10 min. Finally, certain concentrations of the NaOH and HNO_3_ solutions were used in order to adjust the pH to 9. The mixture solution was left overnight, transferred to a Teflon-lined stainless-steel autoclave, and maintained at 180°C for 12 h. The resulting yellow powder was centrifuged and washed three times in a water and ethanol solution, and then oven dried at 80°C for 6 h. In this study, the obtained yellow BiVO_4_/P25 composite with doping of 0.05, 0.1, 0.15 and 0.2 g is referred to as a-BiVO_4_/P25, b-BiVO_4_/P25, c-BiVO_4_/P25 and d-BiVO_4_/P25, respectively, for comparison purposes.

### 2.4. Analytical characterization

The BiVO_4_ and BiVO_4_/P25 crystalline phases were measured on a SmartLab (3 KW) X-ray diffractometer, with Al K*α* radiation. The morphology and composition of the BiVO_4_ and BiVO_4_/P25 samples were performed using an FEI Quanta 400 FEG scanning electron microscope, along with an FEI Tecnai G2 F20 transmission electron microscope operated at 200 kV. The X-ray photoelectron spectroscopy (XPS) analyses of the BiVO_4_/P25 nanocomposite were carried out using a Thermo ESCALAB 250XI spectrometer. The optical properties of the BiVO_4_/P25 samples were estimated by a Lambda 650 UV–vis spectrophotometer using the BaSO_4_ as reflectance sample. A Brunauer–Emmett–Teller (BET) surface area test was performed at 77 K using a TriStar II 3020 surface area analyser.

## 3. Results and discussion

### 3.1. Crystal structure, morphology and composite characterization

**Figure 1.**
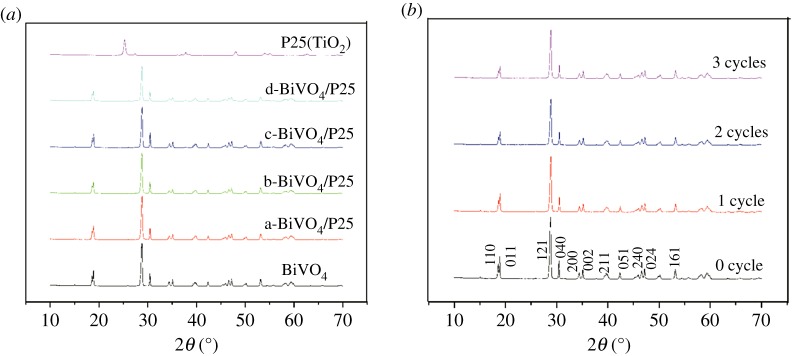
(*a*) X-ray diffraction patterns of the BiVO_4_; a-BiVO_4_/P25; b-BiVO_4_/P25; c-BiVO_4_/P25; d-BiVO_4_/P25 and P25 samples. (*b*) XRD of BiVO_4_/P25 samples after three times reuse to degrade MB solution.

**Figure 2.**
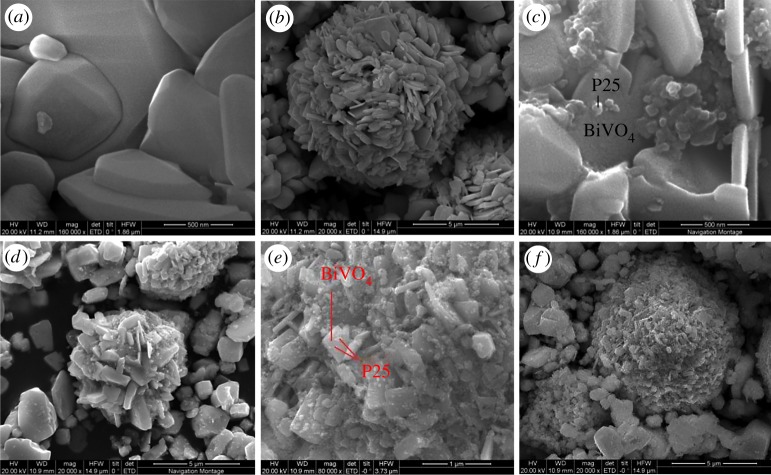
SEM images of the BiVO_4_ (*a*,*b*); BiVO_4_/P25 before degradation (*c*,*d*) and BiVO_4_/P25 after three times reuse to degrade MB solution (*e*,*f*).

**Figure 3.**
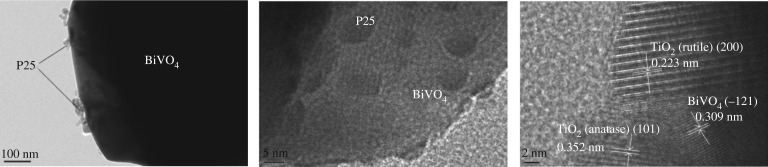
TEM images of the BiVO_4_/P25.

Figure 1*a* details the result of the XRD. The curve of the BiVO_4_ indicates the XRD patterns of the pure BiVO_4_ and its characteristic diffraction peaks at approximately 2*θ* = 18.6°, 18.8°, 28.8°, 30.4°, 35.1°, 39.9°, 42.4°, 46.0°, 46.6°, 47.2°, 50.2°, 53.2°, 58.2° and 59.4° represent the (110), (011), (120), (040), (200), (002), (211), (150), (024), (202), (161), (321) and (123), respectively, of the monoclinic crystal BiVO_4_ according to the JCPDS no. 14-0688 [35]. It was observed that, with the increasing amounts of P25, no diffraction peaks appeared at 2*θ* when compared with the planes of the rutile P25 (JCPDS no. 84-1285) [36]. It was indicated from the curves of the a-BiVO_4_/P25, b-BiVO_4_/P25, c-BiVO_4_/P25 and d-BiVO_4_/P25 that no other peaks were detected. Therefore, no other crystalline phases were observed following the P25 doping, and the doping of the P25 had not changed the crystal structure of the BiVO_4_. Figure 1*b* displayed the XRD patterns of the unused BiVO_4_/P25 samples and BiVO_4_/P25 samples after three cycles for degradation of MB solution. We could know from the picture, as the recycle times increased, the diffraction peaks on the (011) plane, (121) plane and (161) plane weakened, but BiVO_4_/P25 samples of 1 cycle, 2 cycles and 3 cycles still showed the characteristic diffraction peaks at 2*θ* = 18.6°, 18.8°, 28.8°, 30.4°, 35.1°, 39.9°, 42.4°, 46.0°, 46.6°, 47.2°, 50.2°, 53.2°, 58.2° and 59.4°. The results explained that the crystals of BiVO_4_/P25 were not destroyed after repeated recycling and the photocatalyst was stable after reactions. The SEM image detailed in figure 2*a* shows that the pure BiVO_4_ sample exhibited a smooth polyhedral morphology with a thickness of 500 nm. The thickness of 5 µm was observed to be flower-like. Figure 2*c* shows that the P25 nanoparticles were doped on the surface of the BiVO_4_, which made the surface rough with a higher specific surface area than that the single BiVO_4_. This resulted in improved photocatalysis and absorption. Also, the shape was maintained as before the doping process. As can be seen in figure 2*c*,*d* the P25 nanoparticles were found to be deposited on the surface of the BiVO_4_ (040) crystal facet. The P25 solid coating on the surface of the BiVO_4_ could be seen more clearly using a transmission electron microscope. Figure 2*e*,*f* displayed the BiVO_4_/P25 composite after three cycles of degradation of MB solution which revealed that the P25 nanoparticles were discovered on the surface of BiVO_4_ and there was no change in the structure of BiVO_4_/P25. The HRTEM image (the right of figure 3) shows that the lattice fringes of BiVO_4_ /P25 system and the uniform fringes with interval of 0.309 nm were in good agreement with the (−121) lattice plane of monoclinic BiVO_4_, and the interplanar distance of 0.352 and 0.223 nm agrees well with the lattice spacing at (101) plane of anatase TiO_2_ and (200) plane of rutile TiO_2_. Thus, the results confirmed that the heterostructure was formed from P25 and BiVO_4_ [37]_._

**Figure 4.**
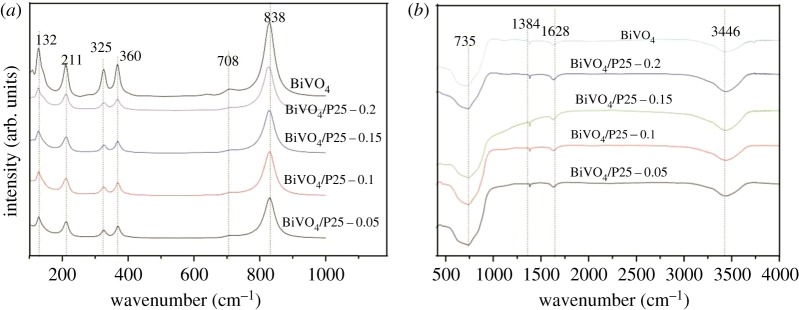
(*a*) Raman spectra of BiVO_4_; a-BiVO_4_/P25; b-BiVO_4_/P25; c-BiVO_4_/P25 and d-BiVO_4_/P25; (*b*) FT-IR spectra of BiVO_4_; a-BiVO_4_/P25; b-BiVO_4_/P25; c-BiVO_4_/P25 and d-BiVO_4_/P25 samples.

The Raman spectrum of the single monoclinic BiVO_4_ and BiVO_4_/P25 samples is presented in figure 4*a*. As shown in figure 4*a*, the Raman bands nearly at 132, 211, 325, 360, 708 and 838 cm^−1^ were due to vibrational modes of monoclinic BiVO_4_ [38], the Raman bands of pure BiVO_4_ and BiVO_4_/P25 samples have shown the same vibration peak, and with the increasing P25 nanoparticles the intensity peak decreasing. Figure 4*b* shows the FT-IR spectrum of single BiVO_4_ and BiVO_4_/P25 samples. The broad absorption at 3446, 1628 and 1384 cm^−1^ corresponds to H–O–H band of the adsorbed water molecules [39]. The absorption band at 735 cm^−1^ in the FT-IR spectra of those samples was due to the asymmetrical and symmetrical stretching vibrations of $\text{V}{\text{O}}_{4}^{3-}$ a tetrahedron in monoclinic BiVO_4_ [40].

**Figure 5.**
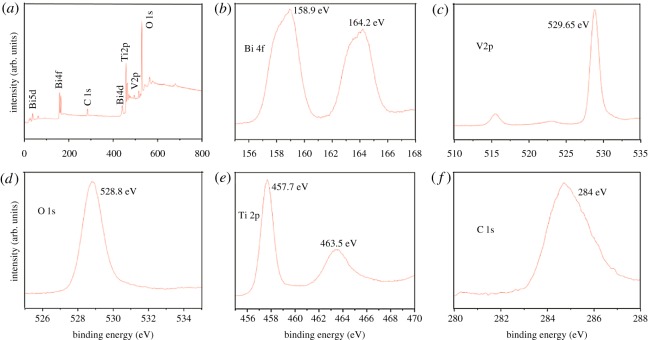
XPS spectra of sample BiVO_4_/P25: (*a*) total survey spectrum; (*b*) Bi4f XPS core level spectrum; (*c*) V2p XPS core level spectrum; (*d*) O1s XPS core level spectrum; (*e*) Ti2p XPS core level spectrum; (*f*) C1s XPS core level spectrum.

The XPS spectra detailed in figure 5 illustrates the main surface elements of BiVO_4_/P25, which indicated that Bi, V, O, Ti and C appeared in the BiVO_4_/P25 composites. The C1s were mainly the remains of the alcohol which was used during the washing process. As can be seen in figure 5, the typical orbital of the Bi4f_7/2_ and Bi4f_5/2_ peaks (figure 5*b*) [41], and the V2p_3/2_ and V2p_1/2_ peaks (figure 5*c*) were observed with the peak locations of 158.9, 164.2, 517.2 and 529.6 eV, respectively, which were determined to be in close accordance with the Bi^3+^ and V^5+^ peaks in the monoclinic scheelite BiVO_4_.

### 3.2. Optical analysis

**Figure 6.**
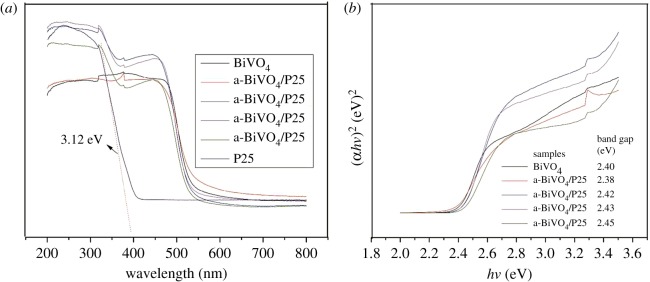
(*a*) UV–vis diffuse reflectance spectra of samples BiVO_4_; a-BiVO_4_/P25; b-BiVO_4_/P25; c-BiVO_4_/P25; d-BiVO_4_/P25; (*b*) band gap of BiVO_4_; a-BiVO_4_/P25; b-BiVO_4_/P25; c-BiVO_4_/P25; d-BiVO_4_/P25.

Figure 6*a* shows the UV–vis diffuse reflectance spectra of the pure BiVO_4_ and BiVO_4_/P25 samples. The band gap (*E_g_)* for a direct band gap semiconductor can be determined from the plots of (*ahv*)^2^ as a function of *hv* (figure 6*b*) originating from the equation [12]: *ahv* = *A* (*hv − E_g_*)^1/2^ . We calculated that the *E_g_* of BiVO_4_, a-BiVO_4_/P25, b-BiVO_4_/P25, c-BiVO_4_/P25 and d-BiVO_4_/P25 were 2.4 eV, 2.38 eV, 2.42 eV, 2.43 eV and 2.45 eV, respectively. The results revealed that the absorption edges of the BiVO_4_ were approximately at 540 nm (figure 6*a*). When the P25 was doped on BiVO_4_, a red shift was observed. However, with the increases in the added P25, the light absorption threshold of the BiVO_4_/P25 gradually blue shifted, which indicated that the BiVO_4_ which had been doped with 0.15 g of P25 did not broaden the absorption of the visible light when compared with the pure BiVO_4_, and certain blue shifts may have been inhibited by the recombination of the photon-generated carrier [42]. The surface area of the BiVO_4_/P25 was determined to be 1.2780 m^2^ g^–1^, while that of the pure BiVO_4_ was 1.0043 m^2^ g^−1^. The BET surface area of the BiVO_4_/P25 was found to be larger than that of the non-doped BiVO_4_, which clearly indicated that the P25 doping could enhance the BET surface area to a great degree.

**Figure 7.**
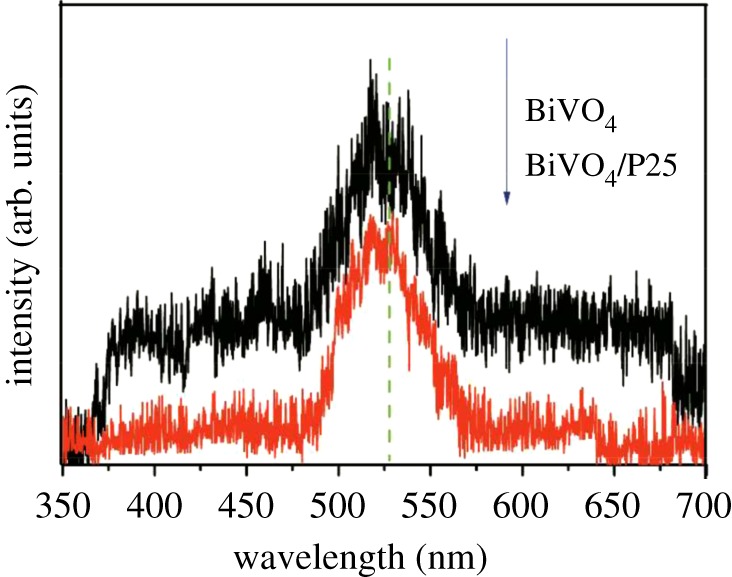
Photoluminescence (PL) spectra of BiVO_4_ and BiVO_4_/P25 samples.

Photoluminescence spectra were often employed to study the migration, transfer and trapping of the photogenerated electron–hole pairs. In semiconductor particles, less intense photoluminescence (PL) usually indicated better separation efficiency of e^−^/h^+^ pairs, thus better photocatalytic activity [43]. Figure 7 shows the PL spectra of the composites excited at a wavelength of 325 nm. It could be seen from the figure that the emission peaks were similar in shape and the maximum intensity was near 530 nm. The peak value of BiVO_4_ was the higher than that of BiVO_4_/P25, which indicated that P25 doping could prevent photoelectron and photogenerated hole from recombination to some extent. That also supported the conclusions from the UV–vis analysis.

### 3.3. Photocatalytic properties

**Figure 8.**
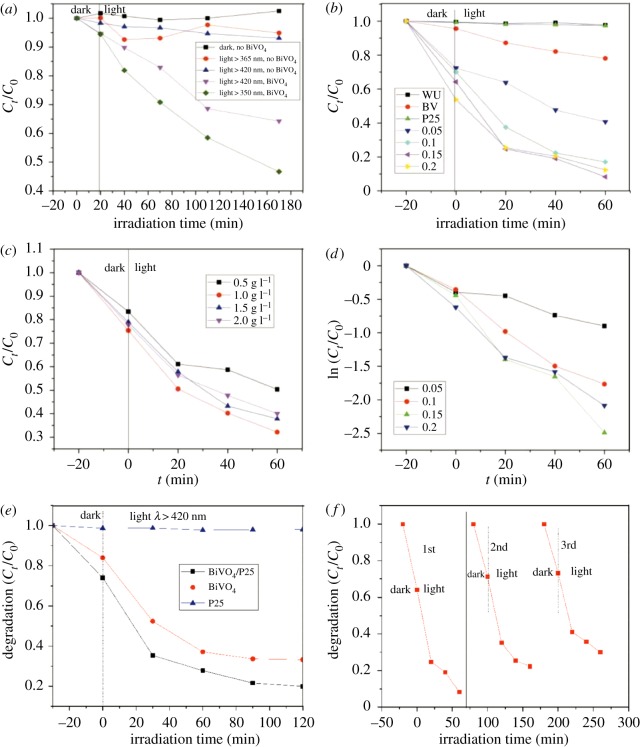
(*a*) Degradation of MB solution under different conditions; (*b*) degradation of the MB solution under visible light irradiation in the presence of the a-BiVO_4_/P25 (0.05 curve); b-BiVO_4_/P25 (0.1 curve); c-BiVO_4_/P25 (0.15 curve); and d-BiVO_4_/P25(0.2 curve); (*c*) degradation of the MB solution with different concentrations of BiVO_4_/P25; (*d*) kinetic curve of the degradation of methylene blue by the a-BiVO_4_/P25 (0.05 curve); b-BiVO_4_/P25 (0.1 curve); c-BiVO_4_/P25(0.15 curve); and d-BiVO_4_/P25 (0.2 curve); (*e*) degradation of tetracycline; (*f*) degradation of methylene blue by three cycles.

The photocatalytic performances of the BiVO_4_ and the BiVO_4_/P25 composites were evaluated by the degradation efficiency of the MB solution and tetracycline solution under the visible light illumination using a xenon lamp as the light source. In the following pictures, the ‘*C*_0_’ represents the initial absorbance, ‘*C_t_*' represents the absorbance after *t* times irradiation, using the (1 − *C_t_*/*C*_0_)% to calculate its degradation rate of MB solution and tetracycline solution. Figure 8*a* details the degradation of the MB solution under different conditions. The results indicated the MB solution almost did not degrade when in dark conditions, and without any catalyst. The degradation under visible light was observed to be lower than with ultraviolet light in the presence of the BiVO_4_. Figure 8*e* illustrates the degradation of tetracycline solution (20 ug l^−1^) by BiVO_4_/P25, BiVO_4_ and P25 composites (the dosage amount was 0.1 g/30 ml) under visible light irradiation. The curves display that the degradation and absorption ability of tetracycline was BiVO_4_/P25 > BiVO_4_ > P25, and we concluded that a suitable amount of P25 doping could enhance the photocatalytic performance and absorption ability of BiVO_4_. Figure 8*b* displays that the a-BiVO_4_/P25 (0.05 curve); b-BiVO_4_/P25 (0.1 curve); c-BiVO_4_/P25(0.15 curve) and d-BiVO_4_/P25 (0.2 curve) composites showed strong adsorption capacities. Also, it was observed that the c-BiVO_4_/P25 showed superior adsorption capacity and photocatalytic abilities when compared with the a-BiVO_4_/P25, b-BiVO_4_/P25 and d-BiVO_4_/P25. Meanwhile, the WU, BV and P25 curves showed the degradation of the MB solution by no catalytic, pure BiVO_4_ and P25 under visible light irradiation. The most effective dosage amount of the c-BiVO_4_/P25 was determined to be 1.0 g l^−1^ (figure 8*c*). This showed that the dark conditions and P25 almost did not degrade the MB solution. Moreover, the degradation of the pure BiVO_4_ to the MB was very small, which indicated that the loading of the P25 could improve the photocatalytic performance of the BiVO_4_. The degradation kinetic curves of the MB solution by the BiVO_4_/P25 (figure 8*d*) detail the degradation of methylene blue by the four different P25-doped BiVO_4_/P25 particles.

In a word, the degradation rate of the BiVO_4_/P25 was the fastest when the doping amount of the P25 was 0.15 g. With the increases in the P25 doping, the adsorption of the methylene blue on the composite catalyst BiVO_4_/P25 was observed to be improved. However, the photocatalytic degradation ability had been weakened, which indicated that excessive P25 doping, and small amounts of P25 doping, were not conducive to improving the photocatalytic performance of the BiVO_4_. The excessive amount of P25 doping tended to affect the absorption of light by the BiVO_4_, and the poor effects of re-using the P25 doping was not conducive to the improvement of the performance of the BiVO_4_/P25 [44]. The optimum doping amount of the P25 was determined to be 0.15 g under this study's experimental conditions, which was found to effectively improve the photocatalytic ability of the BiVO_4_, as well as maintaining good catalytic performances after recycling three times. As figure 8*f* shows, its photocatalytic activity was weakened a little, but still maintains high stability after three cycles.

### 3.4. Photocatalytic mechanism

Based on the Results and discussion, heterostructure formed in BiVO_4_/P25 system played an important role in the efficient separation of photo-induced charge. The CB and VB potentials of BiVO_4_ and P25 at the point of zero charge could be calculated by the following empirical equations (3.1) and (3.2) [45]:

3.1 $$E_{\mathrm{VB}}=X-E^{e}+0.5E_{g}$$

and

3.2 $$E_{\mathrm{CB}}=E_{\mathrm{VB}}-E_{g},$$

where *E*_VB_ is the VB edge potential; *X* is the electronegativity of the semiconductor, which was the geometric mean of the electronegativity of the constituent atoms (*X* value of BiVO_4_ and P25 are approximately 6.04 and 5.81 eV, respectively); *E*^e^ is the energy of free electron on the hydrogen scale (approximately 4.5 eV); *E*_g_ is the band gap energy of the semiconductor; and *E*_CB_ is the CB edge potential. The band gap energies of BiVO_4_ and P25 are adopted as 2.42 and 3.12 eV, respectively. The *E*_VB_ value of monoclinic BiVO_4_ crystallites and P25 crystallites calculated by equation (3.1), and the *E*_CB_ values of those can be obtained by equation (3.2) [46–48].

**Figure 9.**
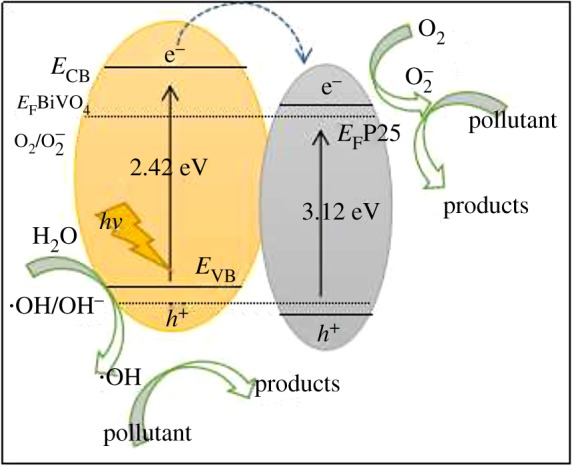
Possible MB hydrogen generation mechanism of BiVO_4_/P25 under visible light conditions.

BiVO_4_ and P25 were the n-type semiconductors. The band gap energy of n-type BiVO_4_ was narrower than that of n-type P25 before any contact between them. When less P25 is doped on the surface of BiVO_4_, a n-N junction was formed on the surface of BiVO_4_/P25 heterostructure [49]. The energy band gap of BiVO_4_/P25 heterojunction catalyst after thermodynamic equilibrium is shown in figure 9. And the BiVO_4_ was considered as an intrinsic semiconductor, so the Fermi level in BiVO_4_ lies in the middle of CB and VB, which was approximately equal to 1.6 eV. Generally speaking, the CB potential (*E*_CB_) of n-type semiconductor because of the more negative potential (approx. 0.1–0.2 V) than the flat-band potential, Fermi level in P25 is −0.1 eV. When these two types of semiconductor materials were closely joined together, the heterostructure photocatalyst BiVO_4_/P25 is formed [50]. At this moment, uniformed the same Fermi level and the system was in equilibrium. After the thermodynamic equilibrium, *E*_F_ turns to be −0.1 eV which was the same as the Fermi level of P25. At the same time, *E*_CB_ and *E*_VB_ decrease from 0.4 to −1.3 eV, 2.8 to −1.1 eV, respectively. During visible light irradiation, electrons on the surface of BiVO_4_ were activated by certain energy photons [51]. Then electrons jumping from VB to CB of the BiVO_4_ had left holes behind in the VB of BiVO_4_. At the same time, the electrons were quickly transferred to the CB of P25 due to the different E_CB_ between BiVO_4_ and P25. Thus, photogenerated electrons can be transferred through more pathways in the system. So that the utilization efficiency of BiVO_4_ on visible light can be improved.

It is widely acknowledged that hydroxyl radicals (•OH) and superoxide anion radicals (O_2_•) play important roles in the photocatalytic oxidation process. Therefore, photoluminescence (PL) technique was employed to study the migration, transfer and trapping of the photogenerated electron–hole pairs in the BiVO_4_/P25 system. From the PL, we could indicate better separation efficiency of e^−^/h^+^ pairs, the holes (h^+^) in the VB of the BiVO_4_ oxidized the H_2_O into a hydroxyl radical (•OH), which had a high oxidizing ability and could effectively oxidize the MB into small products, as well as the end products of CO_2_ and H_2_O [52,53]:

3.3 $$\text{BiV}{\text{O}}_{4}/\text{P25 }+\;\text{pollutant}\to \text{BiV}{\text{O}}_{4}/\text{P}25/\;\text{pollutant}\;(\text{dark absorption}),$$

3.4 $$\text{BiV}{\text{O}}_{4}+\text{hv }\to {\text{e}}^{-}+{\text{h}}^{+}(\lambda >420\;\text{nm}),$$

3.5 $${\text{h}}^{+}+{\text{H}}_{2}\text{O}\to ∙\text{OH, }$$

3.6 $${\text{e}}^{-}+{\text{O}}_{2}\to {\text{O}}_{2}{∙}^{-},$$

3.7 ∙OH + pollutant→ products (main oxidizing process)

3.8 $$\;\text{and}\;{\text{O}}_{2}{∙}^{-}+\;\text{pollutant}\to \;\text{products}.$$

## 4. Conclusion

In summary, in this study, the BiVO_4_/P25 composite which had been prepared by a one-step hydrothermal method displayed a good visible light catalytic capacity and a superior absorption performance. The improved absorption ability contributed to a rough surface and large surface area due to the nanoparticle P25 adherence to the surface of the BiVO_4_ plane. Also, the 0.1 g BiVO_4_/P25 nanocomposites displayed a red shift. It was determined that the addition of P25 did not change the crystals and morphology of the BiVO_4_.

## Data accessibility

Our data are deposited at Dryad Digital Repository: http://dx.doi.org/10.5061/dryad.8r7cn62 [54].
